# Recipes for use in DBPCFC and the importance of blinding

**DOI:** 10.1186/2045-7022-1-S1-S17

**Published:** 2011-08-12

**Authors:** Berber Vlieg-Boerstra, Carina Venter

**Affiliations:** 1 Emma Children's Hospital AMC, Pediatric Respiratory Medicine and Allergy, Amsterdam, Netherlands; 2 University of Portsmouth, Portsmouth, UK

## 

One of the crucial features of double-blind, placebo-controlled food challenge test (DBPCFC) is sufficient blinding of the allergenic food. Blinding is important to avoid any bias during the food challenge. Neither the patient nor the health care professionals involved in the test should be able to identify when the placebo or active test food are being administered. Only if this is guaranteed, the test is actually performed in a double blind fashion. Therefore, without the availability of blinded challenge materials (recipes), true double-blind test conditions cannot be achieved. The validation of challenge materials for adequate blinding can be achieved by sensory testing. Sensory testing should preferably be performed in a professional food laboratory using professional panellists. The second best option is to base the validation on the assessments of volunteers. There is a need for a broad range of validated recipes for the most common allergenic foods in order to cover all age groups, all allergenic foods, to meet the preferences and dislikes of fussy eaters and to take into account cultural eating habits. During the session the availability of validated DBPCFC recipes will be discussed. Participants will be given to chance to participate in a sensory tasting session.

